# Clinical outcome of various metformin treatments for women with polycystic ovary syndrome

**DOI:** 10.1002/rmb2.12026

**Published:** 2017-04-04

**Authors:** Toshiya Matsuzaki, Altankhuu Tungalagsuvd, Takeshi Iwasa, Munkhsaikhan Munkhzaya, Kiyohito Yano, Yiliyasi Mayila, Takako Tokui, Rie Yanagihara, Sumika Matsui, Takeshi Kato, Akira Kuwahara, Minoru Irahara

**Affiliations:** ^1^ Department of Obstetrics and Gynecology Graduate School of Biomedical Sciences Tokushima University Kuramoto Japan

**Keywords:** clomiphene, metformin, ovulation induction, polycystic ovary syndrome

## Abstract

**Aim:**

Polycystic ovary syndrome (PCOS) is an ovulatory disorder and insulin resistance and diabetes are involved in its pathophysiology. Metformin, an anti‐diabetic agent, has been reported to be useful to induce ovulation.

**Methods:**

Metformin treatment was classified into four types: (1) clomiphene–metformin combination treatment for clomiphene‐resistant patients; (2) clomiphene–metformin combination for clomiphene‐sensitive patients; (3) clomiphene–metformin combination for naïve patients; and (4) metformin monotherapy. The patients underwent physical, endocrinological, and clinical examinations for their ovulation rates, pregnancy rates, and follicular development.

**Results:**

The ovulation rates, pregnancy rates, and single follicular development were not significantly different among the clomiphene–metformin combination treatment groups. In the Body Mass Index (BMI) subanalysis, the pregnancy rate was higher in the BMI≥30 kg/m^2^ group than in the other three groups with a BMI of ≤30 kg/m^2^ in both cycles and cases. The ovulation rates and pregnancy rates were significantly higher in the group with a fasting insulin of ≥15 μU/mL than in the groups with a fasting insulin of <15 μU/mL in both cycles and cases.

**Conclusion:**

Clomiphene–metformin combination treatment appears to be useful, at least for clomiphene‐resistant patients, and a BMI of >30 kg/m^2^ and a fasting insulin of ≥15 μU/mL appear to be predictors of a good result with this treatment.

## 1 Introduction

Polycystic ovary syndrome (PCOS) is an ovulatory disorder that is characterized by hyperandrogenism, infertility, and anovulation that is seen in 6%‐10% of women of reproductive age. Insulin resistance, diabetes, and hypertension are involved in the pathophysiology of PCOS,^1^ and metformin, an anti‐diabetic agent, has been reported to be useful for inducing ovulation in patients with PCOS. The Japan Society of Obstetrics and Gynecology, Reproductive Endocrine Committee has created treatment guidelines for PCOS and if patients with PCOS fail to ovulate by the use of clomiphene, then clomiphene plus metformin combination therapy is positioned as a treatment option.^2^, ^3^, ^4^ In a meta‐analysis, clomiphene–metformin combination therapy in clomiphene‐resistant patients was more effective than clomiphene‐alone therapy for ovulation,^5^ pregnancy, and live birth rates.^6^ However, the therapeutic effect of metformin in reproduction is not established, as it has been difficult to integrate the large amount of clinical data in one facility because it is not approved by insurance as an ovulation induction agent. This study was carried out to study the usefulness of ovulation induction by various metformin treatments in patients with PCOS.

## 2 Materials and Methods

### 2.1 Participants

This study was a retrospective study of metformin treatments for Japanese patients with PCOS for ovulation induction, using a questionnaire in 18 facilities. The questionnaire was sent to Okayama University, Kurashiki Central Hospital, Kurashiki Medical Center, Okayama Couple's Clinic, Okayama Aiiku Clinic, Yamaguchi University, Yamaguchi Prefectural Grand Medical Center, Saiseikai Shimonoseki General Hospital, Shimane University, Shimane Prefectural Central Hospital, Matsue City Hospital, Hiroshima Prefectural Hospital, Kinutani Women's Clinic, Tottori University, Kochi University, Tokushima University, Sanno Hospital, and Mimuro Women's Clinic. The metformin treatment was classified into four types: (1) clomiphene–metformin combination treatment for clomiphene‐resistant patients; (2) clomiphene–metformin combination treatment for clomiphene‐sensitive patients; (3) clomiphene–metformin combination treatment for naïve patients, who have no treatment history of clomiphene; and (4) metformin monotherapy. Each type was examined individually**.** Among a total of 178 cases with 798 cycles that were collected from the 18 facilities, 16 cases with 318 cycles were excluded as unsuitable for the four treatments. Thus, a total of 162 cases with 480 cycles were analyzed for their ovulation and pregnancy rates. This study was approved by the Institutional Ethical Committee of Tokushima University, Tokushima, Japan.

### 2.2 Treatments

#### 2.2.1 Clomiphene–metformin combination for the clomiphene‐resistant patients

A total of 80 clomiphene‐resistant patients with PCOS with 221 cycles underwent clomiphene–metformin combination treatment. The ovulation rates per cycle and per case, pregnancy rates per cycle and per case, and the single follicular development rate were analyzed. In addition, subanalyses were carried out according to the Body Mass Index (BMI) (n=70), homeostasis model assessment‐insulin resistance (HOMA‐IR) (n=39), and fasting insulin (n=39) levels. Seventy patients who had BMI data were divided into four subgroups: lean (BMI<18.5 kg/m^2^) (n=9), normal (BMI=18.5‐25.0 kg/m^2^) (n=30), overweight (BMI=25.0‐30.0 kg/m^2^) (n=15), and obese (BMI>30.0 kg/m^2^) (n=16). Thirty‐nine patients who had HOMA‐IR data were divided into four subgroups: HOMA‐IR<0.8 (n=6), HOMA‐IR=0.8‐1.6 (n=10), HOMA‐IR=1.6‐2.5 (n=6), and HOMA‐IR>2.5 (n=17). Thirty‐nine patients who had fasting insulin data were divided into two subgroups: <15 μU/mL fasting insulin (n=29) and ≥15 μU/mL fasting insulin (n=10).

#### 2.2.2 Clomiphene–metformin combination for the clomiphene‐sensitive patients

The clomiphene–metformin combination treatment was given to 57 patients who failed at least three cycles of clomiphene therapy for ovulation induction. In total, 205 cycles were analyzed as the clomiphene–metformin combination treatment for clomiphene‐sensitive patients. The ovulation rates per cycle and per case, pregnancy rates per cycle and per case, and the single follicular development rate were analyzed. In addition, subanalyses were carried out according to the BMI (n=54) and HOMA‐IR (n=37). Fifty‐five patients who had BMI data were divided into four subgroups: lean (BMI<18.5 kg/m^2^) (n=5), normal (BMI=18.5‐25.0 kg/m^2^) (n=34), overweight (BMI=25.0‐30.0 kg/m^2^) (n=7), and obese (BMI>30.0 kg/m^2^) (n=8). Thirty‐seven patients who had HOMA‐IR data were divided into three subgroups: HOMA‐IR<1.6 (n=15), HOMA‐IR=1.6‐2.5 (n=8), and HOMA‐IR>2.5 (n=14).

#### 2.2.3 Clomiphene–metformin combination for the naïve patients

“Naïve” patients were defined as having no treatment history of clomiphene. Seven cases without an infertility treatment history with 12 cycles were treated with the clomiphene–metformin combination treatment as the first‐line treatment for ovulation induction. The ovulation rates per cycle and per case and single follicular development rates were investigated.

#### 2.2.4 Metformin monotherapy

There were 18 cases with 42 cycles of metformin monotherapy. The indications for this therapy were clomiphene resistance (no ovulation) in six cases with 21 cycles, the first choice for ovulation induction in five cases with 10 cycles, habitual abortion in four cases with seven cycles, no pregnancy by clomiphene in an ovulated cycle in one case with one cycle, and a high HOMA‐IR in two cases with three cycles. Fifteen cases were subanalyzed by HOMA‐IR: <1.6 (n=3), 1.6‐2.5 (n=5), and >2.5 (n=7).

### 2.3 Pregnancy and infant outcomes

The outcomes of 64 pregnancies and 29 infants, abortion rates, multiple pregnancy rates, birth weights, birth weeks, and infant anomalies were studied.

### 2.4 Side‐effects

The side‐effects were studied in each treatment group and compared between the clomiphene–metformin combination treatments and the metformin monotherapy.

### 2.5 Dose of metformin, definition of clomiphene resistance, and ovulation rate per case

The usage of metformin and its duration were determined by each facility. Clomiphene resistance was defined as no ovulation with 100 mg of clomiphene, or at least two cycles. The occurrence of ovulation per case with multiple treatment cycles was determined when ovulation was observed in at least half of the treatment cycles.

### 2.6 Statistical analysis

A subanalysis by BMI and HOMA‐IR with treatments 1, 2, and 3 were done by using the chi square test and the Bonferroni post hoc test. A subanalysis by fasting insulin with treatment 1 was done by using the chi square test. In all the analyses, *P*<.05 was considered to be significant.

## 3 Results

### 3.1 Clomiphene–metformin combination treatment for the clomiphene‐resistant patients

**Figure 1:**
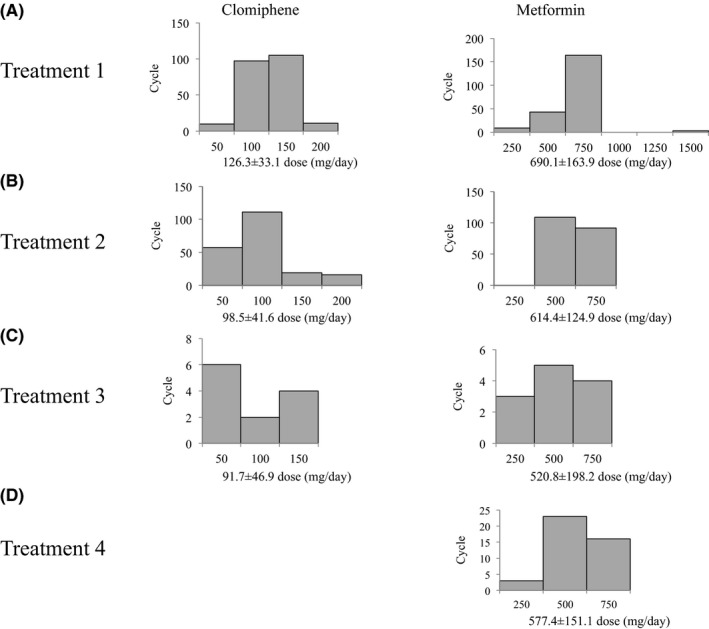
Histogram of the drug dosages with each treatment. A, Treatment 1: clomiphene–metformin combination treatment for the clomiphene‐resistant patients. B, Treatment 2: clomiphene–metformin combination treatment for the clomiphene‐sensitive patients. C, Treatment 3: clomiphene–metformin combination treatment for the naïve patients, and D, Treatment 4: metformin monotherapy. The data are expressed as the means±SD

**Figure 2:**
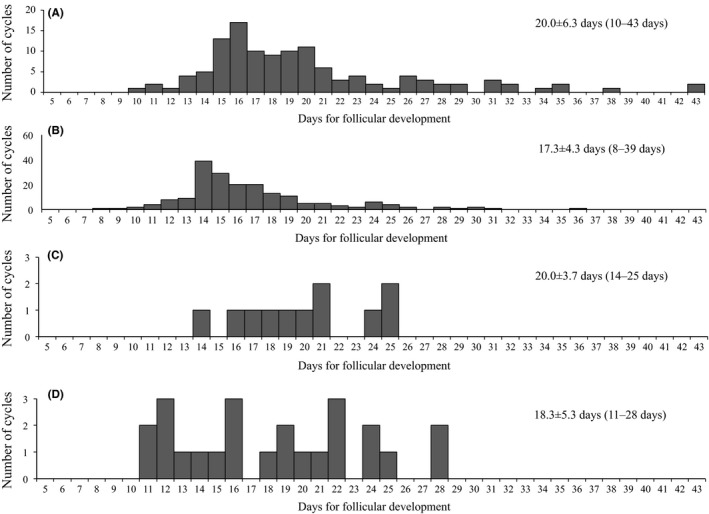
Number of days for follicular development with each treatment (from the first day of the cycle to the day when the follicular diameter reached 18 mm). A, Treatment 1: clomiphene–metformin combination treatment for the clomiphene‐resistant patients. B, Treatment 2: clomiphene–metformin combination treatment for the clomiphene‐sensitive patients. C, Treatment 3: clomiphene–metformin combination treatment for the naïve patients, and D, Treatment 4: metformin monotherapy

**Figure 3:**
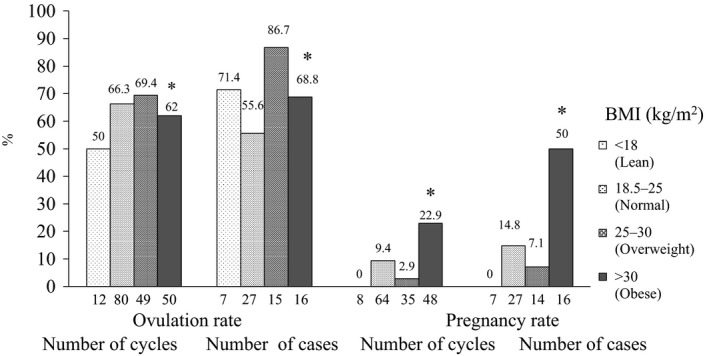
Ovulation rates and pregnancy rates with the clomiphene–metformin combination treatment for the clomiphene‐resistant patients (Treatment 1), according to the Body Mass Index (BMI). *P<.05 vs BMI=18.5–25 kg/m^2^ and BMI=25–30 kg/m^2^

**Figure 4:**
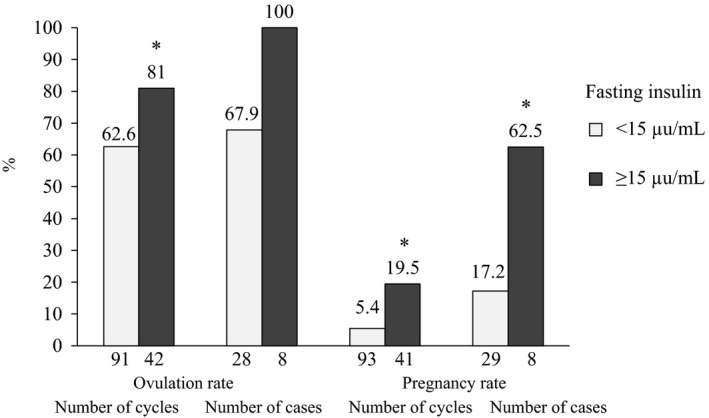
Ovulation rates and pregnancy rates with the clomiphene–metformin combination treatment for the clomiphene‐resistant patients (Treatment 1) by fasting insulin level. *P<.05 vs <15

The mean clomiphene dose was 126.3±33.1 mg/d (mean ± standard deviation [SD]). The clomiphene doses were 150 mg/day (47.5%, 105/221) and 100 mg/d (43.8%, 97/221), both of which accounted for 90.6% of all the treated cycles (Table 1, Figure 1). The mean metformin dose was 690.1±163.9 mg/d. The metformin doses were predominantly 750 mg/day (74.9%, 164/219) in all the cycles. Most of the cycles for the metformin‐treated patients (85.8%, 169/197) were treated with metformin continuous administration and the others (14.2%, 28/197) were treated with short‐term metformin administration from the fifth day of the cycle to the day of the human chorionic gonadotropin (hCG) injection. The ovulation rate was 65.1% per cycle and 69.9% per case and single follicular development was achieved in 81.1% of the cycles (Table 2, Figure 2). The pregnancy rate was 9.9% per cycle and 21.9% per case. In the BMI subanalysis, the pregnancy rate was higher in the obese group than in the other three groups in both cycles and cases (Figure 3). The ovulation rates and pregnancy rates were significantly higher in the group with a fasting insulin of ≥15 μU/mL than in the group with <15 μU/mL fasting insulin in both cycles and cases (*P*<.05) (Figure 4). In addition, the ovulation rate per cycle was 73.9% in the HOMA‐IR<0.8 group, 65.5% in the HOMA‐IR=0.8‐1.6 group, 50.0% in the HOMA‐IR=1.6‐2.5 group, and 72.3% in the HOMA‐IR≥2.5 group; there was no significant difference among the groups. Furthermore, the pregnancy rate per cycle was 5.6% in the HOMA‐IR<0.8 group, 9.1% in the HOMA‐IR=0.8‐1.6 group, 10.0% in the HOMA‐IR=1.6‐2.5 group, and 16.1% in the HOMA‐IR≥2.5 group; there was no significant difference among the groups, although the high HOMA‐IR group tended to have a higher pregnancy rate than the others.

**Table 1:** Background of the participants by treatment

Characteristic	C+M for CR	C+M for CS	C+M for naïve	M
Cycle	221	205	12	42
Case	80	57	7	18
Age (years)	29.6±4.0 (21‐38)	31.0±4.5 (22‐43)	29.9±3.7 (26‐36)	33.2±4.7 (25‐40)
Height (cm)	157.9±5.9 (145‐178)	156.1±4.8 (148‐168)	157.7±5.3 (152‐166)	158.8±5.0 (150‐172)
Weight (kg)	62.9±16.6 (40‐105.6)	57.5±13.5 (42‐93)	59.6±17.8 (44‐94.7)	70.5±15.5 (53‐114)
Body mass index (kg/m^2^)	25.2±6.5 (17.3‐45.0)	23.6±5.4 (17.2‐38.7)	23.7±5.8 (18.8‐34.4)	28.0±6.1 (20.9‐46.3)
Rate of lean and normal cases (%)	55.7	72.2	57.1	38.9
	(39/70)	(39/54)	(4/7)	(7/18)
Rate of overweight and obese cases (%)	44.3	27.8	42.9	61.1
	(31/70)	(15/54)	(3/7)	(11/18)
Age of menarche (years)	12.5±1.6 (10‐18)	12.4±1.4 (10‐15)	12.4±1.1 (11‐14)	12±1.4 (10‐15)
Number of gravida	0.6±0.8 (0‐3)	0.8±1.2 (0‐5)	0.0	1.3±1.7 (0‐5)
Number of para	0.4±0.6 (0‐2)	0.3±0.5 (0‐2)	0.0	0.2±0.4 (0‐1)
Fasting glucose (mg/dL)	92.6±10.4 (70‐127)	97.3±16.6 (82‐176)	89.3±6.4 (82‐93)	97.0±17.1 (72‐145)
Fasting insulin (μu/mL)	16.0±32.6 (0.3‐197.8)	10.5±7.1 (2.2‐27.3)	8.2±13.5 (0.34‐23.8)	16.1±23.8 (0.3‐99.3)
HOMA‐IR	4.0±9.0 (0.06‐55.7)	2.7±2.2 (0.5‐8.8)	0.7±0.1 (0.7‐0.8)	4.6±8.7 (0.1‐35.6)
LH (mIU/mL)	9.0±4.7 (1.6‐25.9)	10.8±8.7 (0.4‐42.9)	13.3±9.1 (4.0‐28.7)	6.5±4.0 (1.8‐12.4)
FSH (mIU/mL)	5.9±1.6 (2.4‐13.4)	5.8±2.0 (2.0‐12.1)	5.2±0.9 (4.1‐6.4)	7.6±6.7 (3.9‐32.5)
LH/FSH ratio	1.6±0.9 (0.3‐5.0)	2.2±2.7 (0.1‐17.2)	2.6±1.8 (1.0‐4.9)	1.0±0.7 (0.3‐3.0)
PRL (ng/mL)	13.8±7.9 (3.3‐44.3)	16.5±8.5 (2.7‐41.3)	11.0±2.8 (8.7‐15.3)	11.6±6.8 (4.2‐34.4)
Testosterone (ng/mL)	0.6±0.3 (0.2‐1.3)	1.0±1.6 (0.1‐8.0)	0.6±0.3 (0.4‐1.1)	0.9±0.7 (0.1‐3.1)
DHEA‐S (ng/mL)	1535±546 (494‐2500)	1717±533 (1030‐2510)	2350±710 (1140‐3600)	1990
E_2_ (pg/mL)	63.2±58.8 (9.3‐325.0)	85.2±66.5 (3.1‐185.0)	57.0±32.7 (23.0‐101.0)	40.7±10.4 (25.0‐53.4)

C+M for CR, clomiphene–metformin combination treatment for the clomiphene‐resistant patients; C+M for CS, clomiphene–metformin combination treatment for the clomiphene‐sensitive patients; C+M for naïve, clomiphene–metformin combination treatment for the naïve patients; DHEA‐S, dehydroepiandrosterone sulfate; E_2_, estradiol; FSH, follicle‐stimulating hormone; HOMA‐IR, homeostasis model assessment of insulin resistance; LH, luteinizing hormone; M, metformin monotherapy; PRL, prolactin.

**Table 2:** Clinical outcomes

Characteristic	C+M for CR	C+M for CS	C+M for naïve	M
Per cycle				
Dose of clomiphene (mg/d)	126.3±33.1 (50–200)	98.5±41.6 (50–200)	91.7±46.9 (50–150)	–
Dose of metformin (mg/d)	690.1±163.9 (250–1500)	614.4±124.9 (500–750)	520.8±198.2 (250–750)	577.4±151.1 (250–750)
Ovulation rate (%)	65.1 (138/212)	91 (181/199)	83.3 (10/12)	75 (27/36)
Number of developing follicles	1.3±0.6 (1‐4)	1.4±1.2 (1‐10)	1.6±1.0 (1‐3)	1.0±0.2 (1‐2)
Rate of single follicular development (%)	81.1 (107/132)	76.1 (140/184)	71.4 (5/7)	95.5 (21/22)
Number of days for follicular development	20.0±6.3 (10–43)	17.3±4.3 (8–39)	20±3.7 (14–25)	18.3±5.3 (11–28)
Pregnancy (%)	9.9 (21/212)	16.7 (33/198)	62.5 (5/8)	15.6 (5/32)
Abortion rate (%)	19 (4/21)	15.6 (5/32)	20 (1/5)	40 (2/5)
Stillbirth rate (%)	5.9 (1/17)	0 (0/21)	–	0 (0/1)
Multiple pregnancy rate (%)	0 (0/16)	0 (0/23)	0 (0/2)	0 (0/5)
Incidence of OHSS (%)	0 (0/53)	0 (0/59)	0 (0/3)	0 (0/6)
Side‐effects (%)	5.5	2	0	2.4
	Diarrhea (5), vomiting (2), nausea (1), bad mood (1), liver dysfunction (1), heartburn (1), eyestrain (1), and uncertain (3)^a^	Diarrhea (2), nausea (1), and stomach ache (1)	(0/12)	Drowsiness and general fatigue (1)
	(12/217)	(4/202)		(1/41)
Per case				
Ovulation rate (%)	69.9 (51/73)	94.3 (50/53)	85.7 (6/7)	60.0 (9/15)
Pregnancy rate (%)	21.9 (16/73)	50.9 (27/53)	83.3 (5/6)	33.3 (5/15)

a There is some overlap. C+M for CR, clomiphene–metformin combination treatment for the clomiphene‐resistant patients; C+M for CS, clomiphene–metformin combination treatment for the clomiphene‐sensitive patients; C+M for naïve, clomiphene–metformin combination treatment for the naïve patients; M, metformin monotherapy; OHSS, ovarian hyperstimulation syndrome.

### 3.2 Clomiphene–metformin combination treatment for the clomiphene‐sensitive patients

The mean clomiphene dose was 98.5±41.6 mg/d. The clomiphene doses were 100 mg/d (54.7%, 111/203) and 50 mg/d (28.1%, 57/203), both of which accounted for 82.8% (168/203) of the cycles for which clomiphene was used (n=203) (Table 1, Figure 1). The mean metformin dose was 614.4±124.9 mg/d. The metformin doses were 500 mg/d (54.2%, 109/201) and 750 mg/d (45.8%, 92/201) in the cycles for which metformin was used (n=201). Most of the cycles (99.5%, 193/194) were treated with metformin continuous administration, except for one cycle, which was treated with short‐term metformin administration from the fifth day of the cycle to the day of the hCG injection. The ovulation rate was 91.0% per cycle and 94.3% per case and single follicular development was achieved in 76.1% of the cycles (Table 2). The pregnancy rate was 16.7% per cycle and 50.9% per case. In the BMI subanalysis, the ovulation rate per cycle was 78.9% in the lean group, 93.0% in the normal group, 89.7% in the overweight group, and 97.3% in the obese group; there was no significant difference among the groups. Additionally, the pregnancy rate per cycle was 16.7% in the lean group, 22.0% in the normal group, 10.3% in the overweight group, and 10.8% in the obese group; there was no significant difference among the groups. The ovulation rate per cycle was 87.5% in the HOMA‐IR<1.6 group, 97.0% in the HOMA‐IR=1.6‐2.5 group, and 91.8% in the HOMA‐IR≥2.5 group; there was no significant difference among the groups. The pregnancy rate per cycle was 17.3% in the HOMA‐IR<1.6 group, 6.1% in the HOMA‐IR=1.6‐2.5 group, and 91.8% in the HOMA‐IR≥2.5 group; there was no significant difference among the groups.

### 3.3 Clomiphene–metformin combination treatment for the naïve patients

The mean clomiphene dose was 91.7±46.9 mg/d. The clomiphene doses were 50 mg/d (50.0%, 6/12) and 150 mg/d (33.3%, 4/12), both of which accounted for 83.3% of all the treated cycles (10/12) (Table 1, Figure 1). The mean metformin dose was 520.8±198.2 mg/d. The metformin doses were 500 mg/d (41.7%, 5/12) and 750 mg/d (33.3%, 4/12). Most of the cycles (62.5%, 5/8) were treated with metformin continuous administration and the others (37.5%, 3/8) were treated with short‐term metformin administration from the fifth day of the cycle to the day of the hCG injection. The ovulation rate was 83.3% per cycle and 85.7% per case and single follicular development was achieved in 71.4% of the cycles (Table 2). Although the ovulation rate seemed high, the usefulness of the treatment was not evident because of the small number of cases.

### 3.4 Metformin monotherapy

There were several reasons why metformin monotherapy was performed. The ovulation rates for the cycles and cases were 33% (6/21) in the clomiphene‐resistant cases and 28% (5/10) in the first‐choice cases, while 22% (4/7) of the patients had an habitual abortion (Table 3). Two (11%) patients had a high HOMA‐IR and one of them achieved ovulation by clomiphene, but not pregnancy (6%). The mean metformin dose was 577.4±151.1 mg/d (Table 1). The metformin doses were 500 mg/d (54.8%, 23/42) and 750 mg/d (38.1%, 16/42) (Figure 1). All 42 cycles were treated with metformin continuous administration. The ovulation rate was 83.3% per cycle in the first‐choice cases, whereas it was 60% per cycle and 40% per case in the clomiphene‐resistant patients (Table 3). The pregnancy rate was 14.3% per cycle and 25.0% per case in the first‐choice cases. There was no pregnancy in both the cycles and cases in the clomiphene‐resistant patients. In addition, the ovulation rate per cycle was 0% in the HOMA‐IR<1.6 group, 87.0% in the HOMA‐IR=1.6‐2.5 group, and 85.7% in the HOMA‐IR≥2.5 group. The HOMA‐IR<1.6 group had a significantly lower ovulation rate than did the other groups (*P*<.05). The usefulness of metformin monotherapy was not clear because of the heterogeneous indications and the small number of cases.

**Table 3:** Clinical outcomes of metformin monotherapy by indication

	Clomiphene resistance (no ovulation)	First choice	Habitual abortion	No pregnancy by clomiphene in the ovulated cycle	High HOMA‐IR
Number of cycles	21	10	7	1	3
Number of cases	6	5	4	1	2
Per cycle					
Ovulation rate (%)	60 (12/20)	83.3 (5/6)	100 (7/7)	–	66.7 (2/3)
Number of developing follicles†	1.0±0.0	1.0±0.0	1.0±0.0	2	–
	(n=13)	(n=2)	(n=4)	(n=1)	
Number of days for follicular development^a^	20.2±5.0 (14–28)	21.4±2.5 (19–25)	14.3±3.7 (12–20)	11	11.5±0.7 (11–12)
Pregnancy rate (%)	0 (0/14)	14.3 (1/7)	42.9 (3/7)	(0/1)	33.3 (1/3)
Abortion rate (%)	–	0 (0/1)	66.7 (2/3)	–	0 (0/1)
Per case					
Ovulation rate (%)	40 (2/5)	100 (3/3)	100 (4/4)	–	50 (1/2)
Pregnancy rate (%)	0 (0/4)	25 (1/4)	75 (3/4)	(0/1)	50 (1/2)

a Mean±SD. HOMA‐IR, homeostasis model assessment of insulin resistance.

### 3.5 Pregnancy outcomes

Multiple pregnancies did not occur among the 42 pregnant patients with all metformin treatments. Only one child (3.4%, 1/29) had a cardiac malformation among the cases that were analyzed. With respect to complications of pregnancy in the 31 pregnant women, there were three cases of gestational diabetes mellitus, one case of hypertension, one case of premature rupture of the membranes, and one case of placenta previa (19.3%, 6/31) (Table 4).

**Table 4:** Pregnancy outcomes and infant anomalies in the women who conceived during the metformin‐treated cycles

Outcome	Value
Spontaneous abortion in the first trimester (%)	20.0 (12/60)
Spontaneous abortion in the second trimester (%)	2.4 (1/41)
Stillbirth (%)	2.9 (1/35)
Multiple pregnancy (%)	0.0 (0/42)
Body weight of the infant (g)^a^ (n=29)	3057.8±527.2 (1807‐4190)
Week of delivery a (n=29)	39 weeks±12 days
	(34 weeks, 3 days‐41 weeks, 5 days)
Anomaly in the infant (%)	3.4 (1/29)
	Cardiac malformation (1)
Complication during pregnancy (%)	19.3 (6/31)
	GDM (3), hypertension (1), PROM (1), placenta previa (1)

a Mean±SD. GDM, gestational diabetes mellitus; PROM, premature rupture of the membranes.

### 3.6 Incidence of side‐effects

Side‐effects of the metformin treatments were observed in 3.6% (17/472) of the patients: diarrhea, vomiting, and stomach ache were classified as digestive system dysfunction (Table 5). There was no significant difference in the incidence of adverse effects between the three clomiphene–metformin combination treatment groups and the metformin monotherapy group.

**Table 5:** Side‐effects of the metformin treatment

Treatment	Drug	Side‐effect (%)	Side‐effects
1. C+M for CR	Clomiphene–metformin	5.5	Diarrhea (5), vomiting (2), nausea (1), bad mood (1), liver dysfunction (1), heartburn (1), eyestrain (1), and uncertain (3)^a^
		(12/217)	
2. C+M for CS	Clomiphene–metformin	2	Diarrhea (2), nausea (1), and stomach ache (1)
		(4/202)	
3. C+M for naïve	Clomiphene–metformin	0.0	–
		(0/12)	
4. M	Metformin	2.0	Drowsiness and general fatigue (1)
		(1/41)	
Subtotal of 1, 2, and 3^b^	Clomiphene–metformin	3.7	–
		(16/431)	
Total		3.6	–
		(17/472)	

^a^There is some overlap. ^b^No significant difference.

## 4 Discussion

Insulin resistance is involved in the pathophysiology of PCOS and is more common in obese, rather than in non‐obese, patients with PCOS. Weight loss, exercise, and insulin sensitizers have been proven to be effective to improve the pathophysiology of PCOS, including the ovulatory disorder.

Metformin is an oral hypoglycemic agent of the biguanide type, which inhibits gluconeogenesis in the liver, promotes sugar use in the peripheral tissue, and suppresses sugar absorption in the intestinal tract. If a patient with PCOS is treated with metformin, insulin resistance is improved and the serum insulin level is reduced, which suppresses ovarian androgen production. In 2003, a meta‐analysis demonstrated that metformin had a higher ovulation induction effect than did the placebo in PCOS (odds ratio [OR]=3.88, 95% confidence interval [CI]=2.25‐6.69).^7^, ^8^ The same result has been replicated in another meta‐analysis (OR=1.81, 95% CI=1.13‐2.93) in 2012.^9^ The incidence of multiple pregnancies in metformin‐treated patients seems to be lower than in clomiphene‐treated patients. For example, multiple pregnancies have not occurred with metformin therapy in Italy,^10^ whereas they have occurred in 6.0% of patients in the USA in a randomized, controlled trial^11^ during clomiphene therapy. The metformin treatment was effective in inducing ovulation or recovering the normal menstrual cycle in the lean or normal BMI patients with PCOS.^12^, ^13^, ^14^, ^15^, ^16^, ^17^, ^18^, ^19^, ^20^ Furthermore, some reports have indicated that non‐obese (BMI<30 kg/m^2^) patients with PCOS were more likely to respond to metformin than obese (BMI≥30 kg/m^2^) patients with PCOS due to decreased sex steroids and increased ovulation rates.^12^, ^14^ From the present study, the metformin dose for PCOS in Japan was 750 mg/d. However, 1500‐2500 mg/d is frequently used in the literature. It was reported that 1500 mg/d of metformin was effective in 87.5% (7/8) of patients who did not respond to 750 mg/d of metformin in clomiphene–metformin combination treatment for clomiphene‐resistant patients.^21^ In the present study, high‐dose 1500 mg/d metformin was used in only two cases with three cycles; it is possible that a higher ovulation rate would be obtained by a 1500 mg/d dose for patients who do not respond to 750 mg/d of metformin.

In a meta‐analysis that studied the clinical outcome of ovulation induction in clomiphene‐resistant patients with PCOS, adding metformin treatment showed significantly superior outcomes to clomiphene monotherapy for the ovulation rate (OR=6.82, 95% CI=3.59‐12.96),^22^ pregnancy rate (relative risk [RR]=5.58, 95% CI=2.34‐13.32), and live birth rate (RR=6.44, 95% CI=1.19‐34.90).^6^ It was concluded that adding metformin to clomiphene is extremely effective for patients with PCOS when clomiphene cannot induce ovulation. In Japanese clomiphene‐resistant patients with PCOS, which included many non‐obese patients, the clomiphene–metformin combination treatment showed a high ovulation rate, which was calculated as ≤55.7% by combining several reports.^23^, ^24^, ^25^, ^26^ Therefore, clomiphene–metformin combination treatment seems to be a useful treatment option for clomiphene‐resistant patients with PCOS. A BMI of >25 kg/m^2^ or impaired glucose tolerance and insulin resistance, even in non‐obese patients, would be good indications, considering metformin's metabolic effects. The multiple pregnancy rate and the incidence of ovarian hyperstimulation syndrome have been reported to be lower with this combination treatment than with clomiphene monotherapy or gonadotropin therapy. It was mentioned that metformin monotherapy was more effective in patients who had mild insulin resistance and were overweight than in severe cases.^27^ In a Cochrane Review subanalysis, pregnancy was more easily obtained with metformin monotherapy than with clomiphene monotherapy in patients with a BMI of <30 kg/m^2^ (OR=1.94, 95% CI=1.19‐3.16).^9^ Furthermore, it was suggested that patients with PCOS who were ≥28 years old and patients with visceral fat accumulation‐type obesity have a higher ability to become pregnant with the clomiphene–metformin combination treatment than with a clomiphene–placebo combination therapy.^28^ Additionally, in the Cochrane Review, ovulation occurred more frequently with the clomiphene–metformin combination treatment than with clomiphene monotherapy in clomiphene‐resistant patients (OR=4.86, 95% CI=2.43‐9.74).^9^ However, the factors that are related to the response to clomiphene–metformin combination treatment have not yet been established. In the present study, the obese group (BMI>30 kg/m^2^) had significantly higher pregnancy rates per cycle and per case with the clomiphene–metformin combination treatment in the clomiphene‐resistant patients. In addition, the ≥15 μU/mL fasting insulin group had significantly higher ovulation rates per cycle and pregnancy rates per cycle and per case. It seems that clomiphene–metformin combination treatment is particularly useful for patients with these backgrounds. However, there were some responders among the patients with a BMI of <30 kg/m^2^ or a fasting insulin of <15 μU/mL. The BMI and fasting insulin are not perfect as factors to predict the response to treatment. In addition, the HOMA‐IR was less useful in predicting the responders.

For the pregnancy outcomes, spontaneous abortion in the first trimester occurred in 20% (12/60) and 17.5% (10/57) of the patients by excluding habitual abortion with metformin monotherapy. This incidence is not high, considering that the participants included a relatively large number of obese patients. In the Cochrane Review, the multiple pregnancy rate (OR=0.56, 95% CI=0.18‐1.68) and abortion rate (OR=1.61, 95% CI=1.00‐1.68) were not significantly different between the clomiphene–metformin combination treatment and clomiphene monotherapy groups.^9^ Only one infant had a heart malformation and the anomaly rate seemed to be not very high (3.4%, 1/29 cases). With metformin monotherapy and the clomiphene–metformin combination treatments, all the pregnancies were singletons in the present study. It was reported that the infant malformation rate in the patients who took metformin in the early pregnancy period was not elevated for those patients with PCOS (OR=0.33, 95% CI=0.07‐1.56) or for the patients with diabetes (OR=.85, 95% CI=0.14‐5.11), compared to the normal population in a meta‐analysis.^29^

The side‐effects of metformin were mainly digestive system dysfunction (5.5%, 12/217 cycles), such as vomiting and diarrhea, with a rate that was comparable to that seen in the treatment of diabetes. Lactic acidosis and hypoglycemia are serious side‐effects of metformin that did not occur in the present study. Lactic acidosis is very rare, affecting three out of 10 million persons per year,^30^ which is likely to occur in patients with liver and kidney dysfunction, and such patients would be few among young women receiving fertility treatment.

The clomiphene–metformin combination treatment appears to be useful, at least for clomiphene‐resistant patients, because of higher ovulation and pregnancy rates; a BMI of >30 kg/m^2^ or a ≥15 μU/mL fasting insulin level could be predictors of a good response to such treatment.

## Disclosures

*Conflict of interest*: The authors declare no conflict of interest. *Human and Animal Rights*: All the procedures were followed in accordance with the ethical standards of the responsible committees on human experimentation (institutional and national) and with the Helsinki Declaration of 1964 and its later amendments. Informed consent was obtained from all the patients to be included in the study.
